# Knowledge, Practice and Attitude towards Foot Ulcers and Foot Care among Adults Living with Diabetes in Tobago: A Qualitative Study

**DOI:** 10.3390/ijerph18158021

**Published:** 2021-07-29

**Authors:** Taiwo Maxwell Adeyemi, Tomi Lois Olatunji, Ademola Emmanuel Adetunji, Satwinder Rehal

**Affiliations:** 1 Scarborough Health Centre, Primary Health Care Department, Bacolet Street, Scarborough, Tobago, Trinidad and Tobago; maxwelladeyemi@hotmail.com; 2 Unit for Environmental Sciences and Management, North-West University, Potchefstroom 2520, South Africa; 3 School of Life Sciences, University of KwaZulu-Natal, Durban 4001, South Africa; adetunjiademola@hotmail.com; 4 Department of Life Sciences, The University of Roehampton, London SW15 5PU, UK; satwinder.rehal@roehampton-online.ac.uk

**Keywords:** diabetic foot ulcer, knowledge, foot care, Tobago, practice

## Abstract

Globally, the prevalence of diabetes has risen significantly by 62% over the last ten years. A complication of unmanaged diabetes is diabetic foot ulcer (DFU), which adversely affects the quality of life of individuals with diabetes and inflicts a huge economic burden on the family, government, and health care services. However, this complication is preventable with adequate patient knowledge and practice regarding DFU and foot care. The present study was aimed at assessing the knowledge, attitude, and practice of adults with diabetes on foot ulcers and foot care in Tobago using a qualitative exploratory design. Purposeful sampling technique was used to recruit 20 participants from the lifestyle and diabetes foot clinics of Scarborough Health Centre, Tobago. Telephone interviews were conducted with the use of a semi-structured interview guide. The data obtained from participants were analyzed using thematic content analysis. Four major themes, namely foot ulcer problems, participants’ knowledge on DFU, knowledge on foot care, and practice and attitude of foot care, emerged from the study. The findings from the study revealed that the majority of participants had poor knowledge regarding DFU but exhibited awareness about foot care, especially on foot cleaning and inspection, preventing irritation after washing, appropriate footwear, and not walking barefooted. The participants had good attitudes and practices of foot care despite their poor knowledge of DFU. However, participants reported inadequate health education on DFU and foot care from healthcare personnel. There should be improved health education, information, and communication on DFU and foot care centred and tailored to the understanding of people living with diabetes. This will prevent DFU and reduce the mortality arising from this complication, which is a major target of the sustainable development goals (SDG) in mitigating the burden of non-communicable diseases (NCD) such as diabetes.

## 1. Introduction

Diabetes is one of the major public health problems in the world, responsible for several health problems, including stroke, blindness, heart attack, kidney failure, and lower limb amputation [1]. In 2017, diabetes mellitus resulted in the death of 4 million adults globally [2]. In 2019, an estimated 463 million individuals lived with diabetes, with a prevalence level of 9.3% in adults. The prevalence of diabetes has increased by 62% over the last ten years (2009–2019), and the prevalence is projected to rise to 10.2% and 10.9% by 2030 and 2045, respectively [3]. 

About 80% of the world’s population with diabetes live in developing countries [4]. This is mainly attributed to the epidemiological transition from infectious diseases to chronic non-communicable diseases (NCDs) in the 21st century [5], which in the context of developing countries, including Tobago, is linked with increased urbanization, ageing population, industrialization, and globalization, which results to changes in the diet, increased consumption of alcohol, sugary food, increased tobacco use, physical inactivity, increased level of stress and sedentary lifestyle [6,7].

In the Caribbean region, diabetes represents one of the major health problems, with a 9% prevalence rate and 13.8% mortality among the adult population [8]. From this region, Tobago is characterized by a high prevalence of diabetes. Globally, Tobago is ranked to have the 5th highest diabetes rate and the highest in the western hemisphere [9]. About 13.6% of deaths were attributed to diabetes mellitus and its associated complications [5]. 

The effort to manage the burden of diabetes has, in effect, placed a huge burden on the economic and public healthcare systems in Tobago. In 2004, the total government expenditure on diabetes and other chronic diseases, such as hypertension, cancer, and cardiovascular disease, was 34 million Trinidadian dollars (TT), equivalent to 5,037,366.54 USD. This figure skyrocketed to TT 121.8 million (18,014,098.20 USD) and TT 400 million (59,159,600.00 USD) in 2009 and 2011, respectively, and at present, a total of TT 3.5 billion (517,646,500.00 USD) is spent on the treatment of diabetes [10]. In 2015, the national health expenditure direct cost for diabetes amounted to 2,063,000,000 USD in Trinidad and Tobago [11]. 

Diabetes is usually associated with several other major complications due to high levels of glucose. Diabetic foot ulcer (DFU) is a common and serious complication of diabetes resulting from the concurrent physiological activities of ischemia, neuropathy, and infection, of which neuropathy accounts for over 60% of foot ulcers [12]. Globally, about 15–25% of adults with diabetes develop foot ulcers during their lifetime. Unsuitable footwear and inappropriate toenail trimming increase the risk of developing foot ulcers in adults with diabetes [4]. In the Caribbean, the prevalence and incidence of DFU is 3.7–4.4% and 17.28%, respectively [13]. DFU patients are susceptible to frequent infections, subsequently leading to morbidity and ultimately amputation [14]. In Trinidad and Tobago, 14% of hospitalizations is due to DFU, and in public hospitals, 29% of bed admissions is due to DFU. Depression occurs in 50% of individuals with lower limb amputation, and in two years, 20% of these patients die [9]. Yearly, the government of Tobago spends 85,701,185 USD (amounting to 0.4% of the national gross domestic product) on treating hospitalized people with diabetes with foot infections [15].

Diabetic foot ulcer is preventable with adequate patient knowledge of the disease and constant examination of the foot [12]. However, there is an increasing rate of foot ulcer incidence among people with diabetes in Trinidad and Tobago [16,17]. Over 40% of people with diabetes with foot ulcers lack sufficient knowledge and education on foot care practices [18,19]. The rapid increase in the incidence of foot ulcers requires efficient preventive and management measures in the reduction and care of diabetic foot ulcers. There are no empirical studies on diabetic’s knowledge of foot care and foot care practices with respect to diabetes management in Tobago. There is, in turn, paucity in understanding the knowledge, attitude, and practice towards foot ulcers and foot care among adults with diabetes in Tobago. There is a need, therefore, to fill this gap in empirical studies. This study, therefore, assessed the knowledge, attitude, and practice of adults with diabetes on foot ulcers and foot care in Tobago as a first step in the planning of targeted self-patient-management programmes by health care providers and policy makers.

## 2. Materials and Methods

### 2.1. Design

A qualitative exploratory descriptive design was adopted for the study to explore the knowledge, attitude, and practice of adults with diabetes about foot ulcers and foot care in Tobago. The qualitative study design was used given the paucity of empirical studies on this subject in Tobago. Hence, an exploration from the perspective of adults with diabetes in Tobago is merited to fill the gap in the literature. 

### 2.2. Study Setting

The study setting was the lifestyle clinic and diabetes foot clinic, both located at the Scarborough Health Centre of the Tobago Regional Health Authority.

### 2.3. Study Participants

We recruited 20 adults with diabetes registered at the lifestyle clinic and diabetes foot clinic using a purposive sampling method. 

#### 2.3.1. Inclusion Criteria

The inclusion criteria for the study were:

1. Voluntary consent to participate in the research.
2. Ability to speak, read and understand English.
3. Adults with diabetes aged 20 years and above with foot ulcers of more than four months who are registered at the lifestyle clinic and diabetes foot clinic and come for dressing at the clinics and/or are patients on admission.

#### 2.3.2. Exclusion Criteria

In this study, exclusion criteria were non-diabetics with foot ulcers, patients with serious co-morbidities, patients that do not provide informed consent, and aged patients to whom the interview guide cannot be objectively administered. Additionally, patients below age 20 and those whose foot ulcers were not up to four months old were excluded from the study.

### 2.4. Recruitment of Participants

Participants’ recruitment involved first getting into contact with the Ward Administrator at the lifestyle clinic and diabetes foot clinic of Scarborough Health Centre, who assisted with access to the admission register of patients who visit the clinic. Calls were also made to participants identified by the Ward administrator’s admission register, and they were invited to participate in the study.

### 2.5. Ethics

The research began after the approval to conduct the study was obtained from the University of Roehampton ethics committee. Ethical approvals were also obtained from the Research Ethics Committee (REC) of the Tobago House of Assembly and the General Manager of the primary care facility at the Scarborough Health Centre.

An informed consent form was given to each participant who gave their approval to participate in the study to sign before data collection.

### 2.6. Data Collection

Telephone interviews were conducted with the use of the semi-structured interview guide. Telephone interview is a widely accepted approach in qualitative data collection that allows relational communication between the researcher and the respondents without physical contact. Given that the study was conducted at the onset of the COVID-19 pandemic, and it was impossible to have face-to-face interview sessions with the participants as a result of the COVID-19 protocols in Tobago, it was appropriate to use telephone interviews to have access to the respondents in Tobago. The interview guide consisted of five parts. The first part consisted of questions to obtain the socio-demographic information of the participants; the second part consisted of questions on past and current foot ulcer problems; the third and fourth parts consisted of guiding questions on knowledge of foot ulcers and foot care, respectively; and lastly, the fifth part consisted of guiding questions on attitude and practice regarding foot care and prevention of foot ulcers. Data were collected by taking notes and audio recording, with permission from the participants, to ensure a precise account of the interview for analysis.

### 2.7. Data Analysis

Data were analyzed using thematic content analysis. The content analysis followed the four main procedures in qualitative research analysis, which include decontextualization (data reduction), re-contextualization, categorization (identification of significant patterns and coding), and the compilation (conclusion drawing from data and verification).

## 3. Results

### 3.1. Socio-Demographic Information of Participants

The age of participants ranged from 28 to 71 years, with a mean age of 55.65 years. Of the 20 participants used for the study, 8 were males, and 12 were females. Participants were predominantly Black, with five participants being of Mixed (Indian-Spanish-Chinese) ethnicity. Fifteen of the participants were married, three were singles, one was divorced, and one was separated. The education level for most of the participants (10) was secondary; six participants had tertiary education, while four participants had basic (primary) education. Three of the participants were unemployed, two were self-employed, nine were employed, and six were retired. The monthly income of the participants ranged from 2000 to 15,000 USD. The longest duration of diabetes among the participants was 40 years, while the shortest duration was two years. Only five participants had, at the time, a current foot ulcer problem, and the longest duration of foot ulcer among them was nine years, while the shortest was four months. They all had diabetes before developing foot ulcers. Three participants were on admission at the time of this study, and 17 were outpatients. The majority of the participants (12) do not consume alcoholic drinks, and others consume alcoholic drinks occasionally. Most of the participants are non-smokers (16), while one participant reported smoking occasionally, and three other participants are heavy smokers (10 sticks per day to 2 packs per week). Only two participants had no family history of diabetes. The treatment used for diabetes by most of the participants was injected insulin, while some take medication and control their diets. Check-up with doctors was predominantly done twice a year by the participants. The detailed baseline socio-demographic characteristics of the participants are shown in Table 1.

### 3.2. Emergent Themes

The data obtained from the participants were analyzed using qualitative content analysis. Key themes, including foot ulcer problem, knowledge on foot ulcers, knowledge on foot care, and foot care practice/attitudes, were obtained from the analysis. These key themes generated 35 sub-themes and 14 categories in relation to the themes and the research questions. Table 2 shows the detailed themes, sub-themes, and the categories obtained from the content analysis.

### 3.3. Foot Ulcer Problems

This theme produced seven sub-themes, which include past foot ulcer diagnosis, present foot ulcer diagnosis, cause of foot ulcer/sore, affected leg, the part affected, amputation due to foot ulcer, and the part amputated. Of the 20 participants interviewed, five reported past foot ulcer problems, while five reported current foot ulcer/sore problems.

Three participants with current foot ulcer reported that it was the big toe of their left feet that were affected, and one participant reported that it was the heel of the right foot that was affected. One participant reported foot ulcers on the toe and heel of both the right and left feet, respectively. Only one participant reported having a current foot ulcer on three toes. 

Participants with current and past foot ulcer problems attributed several reasons for the cause of their foot ulcers. The reasons given by participants for the cause of their foot ulcers included cuts, bumps, and cracks on the foot, rubbing the foot tightly, poor blood circulation resulting in gangrene associated with diabetes, whitish cracks, the weight of the body resting on the foot, and some did not know the reason for their foot ulcers.

One of the participants that attributed the cause of foot ulcer to rubbing the foot too tightly said:

*“I went for a check-up at the clinic, and* *when I got to the health centre, my foot was rubbed too tightly, I had big blood, and it caused the sore. It was bad, but it’s healing”.*

(DFUP07)

All the participants reported bloody, water-like, and pus-like discharge from the foot affected, and they all considered the foot ulcer to be severe.

Three participants only reported amputation of their toes and feet resulting from foot ulcers. Of these three participants, one lost a toe on the left foot, another lost three toes on the left foot, and the third participant reported losing his entire left foot.

### 3.4. Knowledge of Diabetic Foot Ulcer

Five sub-themes, including knowledge on foot ulcers, knowledge on the relationship of diabetes with foot ulcers, knowledge on foot ulcer signs, knowledge on diet and eating habits in preventing foot ulcers, and knowledge on regular use of medication, emerged from this theme.

#### 3.4.1. Knowledge of Foot Ulcer

In this study, the majority of the participants (15) reported that they have no information and no knowledge on diabetic foot ulcers. Others simply said “yes” and that the knowledge they had was limited. One of the participants who affirmed having information on diabetic foot ulcers said:

*“* *Well, yes, from time to time, you pick leaflets and other correspondence on foot ulcer at the health centres”.*

(DFUP04)

Another participant who once had a foot ulcer and reported no knowledge of foot ulcers said:

*“No, when I had foot ulcer, it was very difficult because I did not have much information about it”.*

(DFUP15)

#### 3.4.2. Knowledge on the Relationship of Diabetes with Foot Ulcers

Participants’ knowledge of the relationship of diabetes with foot ulcers was very limited. Most of the participants (15) reported that they do not know how diabetes is related to diabetic foot ulcers. The participants that affirmed knowing the relationship of diabetes to diabetic foot ulcers gave varying responses when asked. The responses that were given by the participants on the relationship of diabetes with diabetic foot ulcers included poor circulation due to diabetes, nerve damage, poorly controlled diabetes, and poor foot care.

A participant who attributed poorly controlled diabetes to the development of foot ulcer said:

*“When the diabetes is* *poorly controlled, you get cracks on the feet, and that can lead to a foot ulcer”.*

(DFUP01)

#### 3.4.3. Knowledge on Signs of Foot Ulcer

Participants’ understanding of the signs of foot ulcers was also explored. Of the 20 participants involved in the study, only some participants with past and current foot ulcers could share their knowledge on the signs of foot ulcers. The signs of foot ulcers as reported by the participants included dry skin, swelling of the foot, cracks on the skin, pain, infection, burning and tingling sensation on the foot, and reddish colouration on the feet. 

A participant who reported swelling as the early signs of diabetic foot ulcer said:

*“My foot was swelling, and I realized one day that I woke up, I noticed no skin on top of the toe. The early sign was swelling”.*

(DFUP20)

#### 3.4.4. Knowledge of Diet and Eating Habits in Preventing Foot Ulcers

The majority of the participants had enough knowledge regarding dietary needs as people with diabetes to control their diabetes, prevent foot ulcers, and hasten the healing of the foot ulcer. They reported that eating vegetables, fruits low in sugar, and avoiding junk and sugary foods will help to control diabetes, heal foot ulcers, and prevent future foot ulcers. The knowledge of eating habits to control diabetes, prevent foot ulcers, and heal foot ulcers were acquired from healthcare providers at the clinic. Despite having this knowledge, some participants with foot ulcers reported that they still eat junk and sugary foods. 

A participant mentioned that proper diet and exercise might help in preventing foot ulcers:

*“Regular medication, which is compulsory, and your diet and exercise will prevent it”.*

(DFUP01)

#### 3.4.5. Knowledge on Regular Use of Medication

Most of the participants also demonstrated adequate knowledge of the regular use of medication for their diabetes and foot ulcers. The majority of the participants affirmed that they take their medications regularly and that it helps in keeping the sugar level low and in hastening the healing of the foot ulcer. Some participants mentioned that they were only given antibiotics and foot powder for their foot ulcers. Several effects of not taking medications regularly were stated by the participants, which included foot deterioration leading to amputation, slow healing of the foot ulcer, and feeling miserable and numb.

A participant reported the implication of not taking medications regularly as:

*“Well, when you don’t take your medication regularly, things go bad, the foot may develop deterioration and leads to amputation”.*

(DFUP16)

### 3.5. Knowledge of Foot Care

Twelve sub-themes were generated from the theme. The sub-themes include knowledge on foot cleaning frequency, foot inspection, bathing water temperature, foot cleaning water temperature, washing feet with soap and water, appropriate footwear, frequency of changing socks, appropriate foot cream, cutting of toenails, walking barefooted, preventing foot irritation after washing, health education knowledge on foot care, and foot care knowledge score rate. Generally, in this theme, the level of knowledge on foot care was comparable among participants with diabetic foot ulcers and those without diabetic foot ulcers.

#### 3.5.1. Knowledge of Foot Cleaning Frequency and Foot Inspection

The majority of the participants demonstrated adequate knowledge on how frequently their feet should be cleaned and inspected. Most participants believe that the feet should be cleaned daily, while some said they should be cleaned at least twice a day. On foot inspection, the majority of the participants mentioned that the feet should be inspected every day while bathing. At the same time, some gave different opinions, like three times a week, only when cutting the toenails, and when they feel different sensations in the feet.

A participant when asked how often feet should be inspected said:

*“It should be inspected* *every time you feel dryness on the skin”.*

(DFUP03)

#### 3.5.2. Knowledge of Bathing and Foot Cleaning Water Temperature

The majority of the participants (15) were of the opinion that bathing and foot cleaning water temperature should be warm; some called it lukewarm, some said tepid, and some said not too hot and not too cold. However, the remaining five participants reported that they know that the water used in bathing and cleaning the foot should be warm, but they do not follow that guideline for several personal reasons.

One of the participants who bathes and cleans the feet with cold water said:

*“I feel strange and funny with warm water; I prefer it to be cold in washing my feet”.*

(DFUP08)

#### 3.5.3. Knowledge on Washing the Feet with Soap and Water, Preventing Feet Irritation after Washing, and Appropriate Foot Cream

The participants gave varying responses with regards to washing the feet with soap and water as part of foot care. While the majority of the participants believed and reported that washing with soap would help remove dirt and prevent foot infection, some participants were indifferent about it. In contrast, some participants reported that the feet should not be washed with soap but with other things like Savlon, methylated spirit, and salt.

A participant who reported that washing the foot with soap will help in disinfecting the foot said:

*“Washing with soap and water* *helps to disinfect your feet and its sores”.*

(DFUP03)

The majority of the participants in the study also demonstrated adequate knowledge on how to prevent foot irritation after washing. They reported drying well between toes, applying moisturizing cream, massaging the foot, foot exercises, applying foot powder, and wearing socks to keep the feet dry will help in preventing foot irritation after washing. Drying the foot properly and between the toes is one major component that resonated in the responses of the participants in preventing foot infection after washing, as seen in the response below:

*“Well, after washing, you need to dry your foot and apply foot powder and put in socks to keep your foot dry”.*

(DFUP15)

The participants gave different responses on the appropriate foot cream to use as people with diabetes. Most of the participants (12) reported that moisturizing cream like Cetaphil should be used on the feet after washing. In contrast, others mentioned antifungal cream, antibacterial cream, coconut oil, paraffin oil, olive oil, baby oil, and Vaseline. Some participants reported using the recommended cream as directed by their health caregivers. A participant who reported paraffin oil as the doctor’s recommendation of foot cream said:

*“The doctor* *recommends paraffin oil, but sometimes I just put Vaseline”.*

(DFUP08)

#### 3.5.4. Knowledge on Appropriate Footwear, Socks, and Frequency of Changing Socks

The majority of the participants (90%) demonstrated adequate knowledge of the type of footwear that adults with diabetes should wear. As reported by the majority of the participants, the knowledge on which shoe to wear was obtained from their health caregivers. Generally, the participants reported that people with diabetes should wear very comfortable, soft, soothing, well-padded, and closed-toe shoes. The participants mentioned slippers, sneakers, and special shoes made for people with diabetes as examples of appropriate footwear.

A participant, when asked about the appropriate footwear, said:

*“A diabetic patient should wear soft, comfortable shoes made from breathable material”.*

(DFUP18)

The majority of the participants (14) in this study do not wear socks. However, those who wear socks gave different responses to how frequently the socks should be changed. Some participants who wear socks and those who do not wear socks said socks should be changed every day, while some said it should be changed every other day, and some even said once in a week.

A participant said:

*“Well, I don’t* *wear socks, but if I did, I would say socks should be changed every day and should be cotton”.*

(DFUP01)

#### 3.5.5. Knowledge on the Frequency of Cutting Toenails and Walking Barefooted

With regards to cutting toenails and the frequency, participants had different views. While the majority of participants (14) believed that cutting the toenails should be done every week, a few others reported that cutting the toenails should be done once every three weeks or just when the nails have grown too long. 

Worthy of mention in this sub-theme is the fact that all participants had adequate knowledge with regards to walking barefooted in relation to a foot ulcer. They understood that walking barefooted as people with diabetes is highly detrimental. The majority of the participants believed that walking barefooted exposes the foot to being pierced by any object, leading to a deep cut, foot ulcer, foot infection, and, consequently, amputation of the foot. Two of the participants reported that they had special shoes they wear at the beach to avoid being pierced on the feet. Below is one of the participants’ responses relaying their knowledge regarding walking barefooted in relation to foot ulcers:

*“Walking barefooted can cause more damage to your feet if you have foot ulcers; it can become infected and then lead to amputation”.*

(DFUP01)

#### 3.5.6. Health Education Knowledge on Foot Care and Knowledge Score Rate

In this sub-theme, all the participants reported that they had never attended classes on foot care. Only three participants affirmed reading handouts on foot care; others reported that they never received a handout on foot care to read. One of the participants who reported reading handouts on foot care said:

*“I learn for myself. Yes, I read handouts on everything about diabetes. I learn everything because it relates to me”.*

(DFUP15)

However, most of the participants (16) reported that they received foot care lessons from their doctors. In contrast, the other four participants simply said they receive no information on foot care from their doctors. Although most participants received information on foot care from their doctors, only seven participants rated their foot care knowledge as good, while the others rated their knowledge as limited.

### 3.6. Foot Care Practice/Attitude

Eleven sub-themes emerged from this theme. These included the practice of foot inspection, foot soaking, washing with soap and warm water, drying well between toes, cutting toenails, wearing socks, appropriate footwear, appropriate sock fabric, walking barefooted, inspecting footwear, and buying appropriate footwear size.

In this theme, as reported by the participants, most of the participants had good foot care practices and attitudes, especially in inspecting the foot every day before and after bathing and under the light, washing the foot daily with soap and lukewarm water, drying well between toes, cutting toenails, not walking barefooted, wearing the appropriate footwear and sock fabric, inspecting footwear, and buying the appropriate footwear size. 

The only participant in this study who reported that her husband checks her feet daily when she cannot reach her feet enthusiastically said that:

*“All the time, every day. Sometimes* *I can’t reach my foot, but every day I will ask my husband to massage my feet and inspect every part because I can’t see under my foot”.*

(DFUP15)

Most of the participants reported that they wear soft footwear made from a breathable material, sneakers, or closed shoes when going to work, and they wear slippers and flip-flops while at home. However, two participants reported walking barefooted only when they are indoors in their homes. 

Most of the participants (15) who wear socks reported wearing socks made of cotton. 

On the practice of foot soaking, most of the participants in this study (15) do not practice foot soaking. In contrast, a few others reported soaking of the foot only when doing pedicures.

A participant who reported testing the water temperature and soaking the feet with warm water and salt said:

*“The neuropathologist* *advised me to soak my feet in the night. It is not a daily regime; I’m inclined to do it when I feel the numbness; I use water and salt, and I test the temperature”.*

(DFUP04)

## 4. Discussion

With the ever-increasing burden of diabetes and its related complications, including diabetic foot ulcers, in the Caribbean, with a prevalence and incidence rate of 4.4% and 17.28%, respectively [13], the present study was carried out to assess the knowledge, attitude, and practice of adults with diabetes on foot ulcers and foot care in Tobago. 

In this study, the participants’ population was primarily made up of the Black race (75%), with the other 25% participants being of Mixed race. This is not unexpected, as Tobago is ethnically dominated by the Black race [20]. The age range of participants in this study was 28–71 years. This age range is consistent with the International Diabetes Federation (IDF) report that type 2 diabetes occurs chiefly in patients between 20–79 years of age [21]. Further, the average age of participants in this study was 55.65 years, which is similar to the previously reported mean age (56.9 years) of adults with diabetes in an Eastern Caribbean population study [16]. Type 2 diabetes has been reported to be predominant in older patients, although there are recent reports of its occurrence in adolescent populations [22].

In contrast to the findings of Islam et al. [16] that reported male gender to be most prevalent in people with diabetes, the number of female participants was higher than male counterparts in this study.

The male predominance for diabetic foot ulcers as reported in other studies [4,22] was also seen in this study, with active cases of foot ulcers occurring in four male participants and only in one female participant. The increased risk of developing diabetic foot ulcers in males has been attributed to greater foot pressure, lessened joint mobility, and higher neuropathy prevalence [22,23]. However, with the occurrence of other risk factors for diabetic foot ulcers and neuropathy, both genders are at equal risk of developing diabetic foot ulcers [23].

The majority of participants in this study were married. This high number may account for the assistance received from their spouses with regards to foot care practice, particularly the inspection of the feet. 

Education level, income, and employment status have been identified as risk factors of foot self-care behaviours [24]. The majority of the participants in this study have secondary education, are employed, and are earning. These factors contributed positively to the foot self-care behaviours of the participants, as most of the participants had a good practice of foot care. These findings are consistent with the report of D’Souza et al. [4], where formal education and income were positively correlated with foot self-care practice for adults with diabetes in Oman.

In this study, 95% of the participants reported having a family history of diabetes. This is similar to several reports globally and in the Caribbean that reported that family history of diabetes is associated with an increased risk of developing type 2 diabetes [25,26]. As reported by the American Diabetes Association (ADA), genetics and the environment play a major role in the development of diabetes, and type 2 diabetes has a stronger link with genetics, particularly family history or lineage of diabetes [27]. 

Three out of the five participants with active diabetic foot ulcers reported to be heavy smokers (10 sticks per day to 2 packs per week). This is interesting, as smoking with diabetes has been associated with higher risks for developing severe diabetic circulation. For example, smoking with diabetes has been linked with the reduced flow of blood in the extremities, peripheral neuropathy, and peripheral arterial problems, which can give rise to foot infections, foot ulcers, and amputation [28]. With diabetic foot infections, smoking has also been associated with delayed healing of wounds [28]. This may be why one of the participants with the longest duration (9 years) of foot ulcer in this study has not yet experienced improvement of their foot ulcer.

In this study, the majority of the participants do not consume alcohol, while the others consume alcohol occasionally. Furthermore, only one participant with an active foot ulcer consumes alcohol daily at the time of this study. Consumption of alcohol in people with diabetes has been correlated with an increased risk of developing peripheral neuropathy. This is because alcohol consumption, even in small amounts, alters the glucose control in the blood, thus increasing the risk of neuropathy and delay in wound healing, consequently leading to the development of foot ulcers [28,29]. 

Summarily, in this study, the socio-demographic factors that might be associated with the development of diabetes and foot ulcers are gender (male), lifestyle factors (smoking and alcohol consumption), and family history of diabetes.

According to a prospective observational study reported by Registered Nurses’ Association of Ontario [19], the most common locations of ulceration are the forefoot, the plantar region of the toes, and the hind foot. The findings of this study are in line with this report, as the plantar region of the toes, particularly the big toe, was the site of ulcer for participants with active ulcer cases.

The cause of diabetic foot ulcers is multifaceted, and it occurs mainly from the concurrent effects of peripheral neuropathy, ischemia, and poor glycaemic control (gangrene) [30,31]. The majority of the participants with foot ulcers reported cuts, cracks, dry skin, and gangrene as the cause of their foot ulcers. This may be attributed to peripheral and autonomic neuropathy, as they are mainly associated with high rates of skin breakdown and dry skin, consequently leading to cuts or cracks. The signs of DFU reported by the participants in this study are in line with the clinical signs of DFU reported by Lipsky et al. [32] and the Registered Nurses’ Association of Ontario [19].

In terms of the main outcome on the participants’ knowledge regarding DFU, this study revealed that participants had poor knowledge of DFU, signs of DFU, and the relationship of diabetes with regards to DFU. This was attributed to inadequate information on DFU from their doctors and the unavailability of health education materials, such as leaflets and handouts. In contrast to this study’s findings, Gale et al. [33] reported that their study participants disregarded information on DFU that did not synchronize with their beliefs. Similarly, the John et al. [34] study in India is incongruent with this study. The study attributed low socio-economic status and lack of formal education among the participants as the major factor contributing to the poor knowledge of DFU. Several studies in the United Kingdom and the Caribbean, including Pollock et al. [35], Basu et al. [36], and Gale et al. [33], reported the majority of the participants (up to 95%) in their studies had no clear knowledge, information, and awareness on what diabetic foot ulcers are. The lack of adequate knowledge of DFU was attributed to little or no information being received from health caregivers.

The knowledge of diet as it relates to diabetes and DFU is an important factor in the management of diabetes and DFU. Proper diet has been linked to lowering blood sugar levels and healing wounds [19]. Participants in this study understood the importance of proper diet and regular use of medication with regard to DFU. The findings in the current study are harmonious with the results of Abioye-Kuteyi et al. [37] and Salia [38]. They assessed the dietary knowledge and practices among people living with diabetes in relation to DFU. Their findings revealed that participants had adequate knowledge of diet and its implication on DM and DFU. As alluded to in our findings, knowledge on proper diet was informed by health caregivers’ education, and this was also reported by Abioye-Kuteyi et al. [37] and Salia [38].

On the knowledge regarding foot care, most participants had adequate foot care knowledge, especially on foot cleaning and inspection, preventing irritation after washing, appropriate footwear, and not walking barefooted. In contrast to this finding, Fatima et al. [39] reported that foot care knowledge was unsatisfactory among their study participants, as only 7% had good foot care knowledge. Hasnain and Sheikh [40] reported that most of their study participants demonstrated adequate knowledge regarding the trimming of toenails and walking barefooted in the prevention of DFU. This finding is consistent with the result of the present study. However, Ekore et al. [41] reported that the majority of their study participants had poor knowledge with regards to cutting toenails and walking barefooted. The study reported that only 22.6% of the participants knew that they are not to walk outdoor barefooted, while 2.9% had understanding about cutting the toenails with care.

On the attitude and practice of foot care, most of the participants in this study had good attitudes and foot care practices despite their poor knowledge of DFU. The findings in this sub-theme are comparable to the results of other similar studies. For example, John et al. [34] conducted a “cross-sectional study to assess the knowledge, attitude, and risk of DFU” among diabetic participants in a tertiary hospital in India. It was reported that the majority of the participants had good foot care practices despite their poor knowledge of DFU. The study attributed low socio-economic status and lack of formal education among the participants as the major factor contributing to the poor knowledge of DFU.

Mbisi et al. [42] conducted a descriptive study to assess foot care practices among people with diabetes in five hospitals in Kenya. The study reported moderate foot care practices by calculating the sum of activities practiced by the participants.

Abo deif and Abdelaziz [43] conducted a descriptive exploratory study to determine the level of knowledge towards foot care and practice in Type 1 and type 2 diabetic patients in Cairo, Egypt. The results of the study showed that both categories of participants had desirable knowledge of foot care. However, foot care practice among them was very poor. The authors attributed poor foot care practice among the participants to the dearth of awareness with regards to complications of diabetes that result from poor foot care practices. 

In contrast to the findings of the present study, Kurup et al.’s [44] study on knowledge, attitude and practice towards foot care and DFU among diabetic and non-diabetic participants in a Caribbean country (Guyana) reported that the subjects (people with diabetes) had good knowledge of DFU, as they had adequate information from healthcare givers; however, they had poor foot self-care practices. The study attributed the poor foot self-care attitude and practices to the fact that participants were not suitably motivated to take care of their feet.

Pourkazemi et al. [45], in contrast to the findings of the present study, reported that the majority of the participants (people with diabetes) from the Guilan province in Iran had very poor practice of foot care. The study reported a significant correlation between knowledge on foot care, which was predominantly poor, and the attitude and practice of foot care among the participants. 

Generally, several issues must be addressed that are faced by people with diabetes in the Caribbean region and, in particular, Tobago, thereby making the management of diabetes and its complications challenging. These challenges include (1) dietary issues: the major menus are carbohydrate-based, whereas the healthy foods (fruits and vegetables) are expensive, and the unhealthy junk foods are cheaper; (2) lack of emotional and psychological support in dealing with diagnosis of diabetes mellitus and coping with complications, as in many cases, the pre and post counselling before amputations can be lacking; (3) appropriate footwear and early treatment of foot problems are inadequate; (4) financial burden of medications, monitoring kits, and other support tools for effective care are also challenging; (5) lack of well-coordinated, integrated diabetes care policies; (6) unavailability of all categories of specialist personnel for effective care; (7) poor family support; (8) insufficient training of patient, family, and medical personnel in diabetes care; (9) low levels of education of patients and the general population in diabetes as a disease; and (10) discrimination issues on jobs, at schools, etc., as people with diabetes are often stereotyped.

Overall, based on the findings of this research, it is important that timely interventions and plans that would help in the prevention and management of DFU in Tobago are implemented. In the design and planning of better patient-management education programmes on diabetic foot ulcers in Tobago, it is recommended (for policy) that the government should sponsor the training of nurses and other health caregivers on the delivery of health education talks to people with diabetes. This training should be geared at providing nurses and other health caregivers with adequate knowledge and competencies in delivering theory- and practical-based health education on foot care. Special nurses training and education on foot care have been identified to have an important role in the prevention and management of DFU [46,47]. Furthermore, for practice, it is recommended that healthcare professionals should make certain to give foot health care education regularly to people with diabetes through improved information, education, and communication strategies by creating models such as the health belief model [48]. Infographic leaflets on DFU and foot care should be made available to people with diabetes always. A pictorial-based information leaflet was reported to be an efficient patient education tool in the management and prevention of DFU [49]. Nurses should endeavour to inspect the feet of people with diabetes for possible foot ulcers at all clinic visits. This could be done following the clinical best practice guidelines by RNAO [19].

Additionally, diabetes care and prevention training for people with diabetes, their families, and the community should be organized, and support groups and advocates for people living with diabetes should be encouraged.

In a recent publication by Lung et al. [50], different emerging technologies, including laser Doppler flowmeter for assessing plantar tissue viability and infrared thermography for early detection of plantar tissue inflammation, among others, have been identified. These emerging technologies could help to quantify the risks and early detection of DFU. The government of Tobago could adopt such technologies to aid the prevention and management of DFU in Tobago. 

Finally, it is recommended that further research on the knowledge, attitude, and practice towards foot ulcers and foot care in adults with diabetes in Tobago should include quantitative studies with an adequate sample size to achieve statistically valid results.

## 5. Conclusions

The study aimed to assess the knowledge, attitude, and practice of adults with diabetes regarding foot ulcers and foot care in Tobago to aid in the design and planning of better patient-management education programmes on diabetic foot ulcers in Tobago. The study revealed that general knowledge of DFU among the participants was inadequate. The inadequate knowledge was attributed to limited or no information on DFU from their healthcare providers. However, participants demonstrated good knowledge, attitude, and practice of foot care, such as regular foot cleaning and inspection, preventing irritation after washing, use of appropriate footwear, and not walking barefooted. To improve on the management of DFU among people with diabetes in Tobago, knowledge on DFU and further practice of good foot care can be improved with more targeted information and education communication on DFU and foot care. Doing this would aid in better public and personal health care outcomes as envisaged in the health targets of the Sustainable Development Goals (SDGs).

## Figures and Tables

**Table 1:** Participants socio-demographic profiles.

Participant ID	Age	Sex	Ethnicity	Marital Status	Education Level	Employment Status	Income/Month ($)	Years of Living with Diabetes	Length of Time with DFU (Years)	Admitted Patient	Out-Patient	Alcohol	Family History of Diabetes	Smoking
DFUP01	71	F	Black	Married	Secondary	Unemployed	3000–5999	˃20	NA	No	Yes	No	yes	No
DFUP02	61	F	Black	Married	Tertiary	Retired	˃10,000	10	NA	No	Yes	No	No	No
DFUP03	44	F	Black	Married	Secondary	Employed	˃10,000	5–10	NA	No	Yes	No	Yes	No
DFUP04	65	M	Black	Married	Secondary	Retired	˃10,000	˃10	NA	No	Yes	Occasionally	yes	Occasionally
DFUP05	70	F	Black	Single	Primary	Retired	˃10,000	˃20	NA	No	Yes	No	Yes	No
DFUP06	62	F	Black	Married	Tertiary	Employed	˃10,000	4–5	NA	No	Yes	No	Yes	No
DFUP07	55	F	Mixed	Married	Secondary	Unemployed	˂3000	˃20	NA	No	Yes	No	Yes	No
DFUP08	49	M	Black	Divorced	Primary	Self-employed	˂3000	˃20	9	No	Yes	No	Yes	Yes (10 S/Day)
DFUP09	50	M	Black	Separated	Secondary	Employed	3000–5999	˃10	3	Yes	No	Yes	Yes	Yes
DFUP10	28	M	Black	Single	Tertiary	Employed	˃10,000	˃10	NA	No	Yes	No	yes	No
DFUP11	55	F	Mixed	Married	Tertiary	Employed	˃10,000	˃10	NA	No	Yes	Occasionally	yes	No
DFUP12	59	M	Black	Married	Secondary	Self-employed	˃10,000	4	NA	No	Yes	No presently, but yes before diabetes	Yes	No
DFUP13	30	F	Black	Single	Secondary	Employed	3000–6000	9	NA	No	Yes	sometimes	Yes	No
DFUP14	43	M	Mixed	Married	Secondary	Employed	˃10,000	6	NA	No	Yes	Not really	Yes	No
DFUP15	68	F	Mixed	Married	Secondary	Unemployed	˂3000	25	5	No	Yes	No	Yes	No
DFUP16	67	F	Black	Married	Primary	Retired	3000–6000	40	NA	No	Yes	Not at all	No	No
DFUP17	63	F	Black	Married	tertiary	Employed	˃10,000	5	NA	No	Yes	Occasionally	Yes	No
DFUP18	46	M	Black	Married	Tertiary	Employed	˃10,000	20	NA	No	Yes	No	Yes	No
DFUP19	58	F	Mixed	Married	Secondary	Retired	˂3000	6	4 Months	Yes	No	No	Yes	No
DFUP20	69	M	Black	Married	Primary	Retired	5000	2	1	Yes	No	No	Yes	Yes (2 Pck/Week)

**Table 2:** Index table showing the key themes, sub-themes, and category from the content analysis.

Themes	Sub-Themes	Category
Knowledge on diabetic foot ulcer	- Knowledge on foot ulcers	
	- Knowledge on the relationship between diabetes and foot ulcers	
	- Knowledge on foot ulcer signs	
	- Knowledge of diet and eating habits in preventing foot ulcers Knowledge on regular use of medication	
Knowledge on foot care	- Knowledge on foot cleaning frequency	Daily/Every other day/Once a week
	- Knowledge on foot inspection	Personal inspection/Inspection by spouses or others
	- Knowledge of bathing water temperature	
	- Knowledge on foot cleaning water temperature	
	- Knowledge on washing feet with soap and water	
	- Knowledge of appropriate footwear	
	- Knowledge of the frequency of changing socks	Daily/Every other day/Once a week
	- Knowledge on appropriate foot cream Knowledge on cutting of toenails and frequency	Personal cutting of toenail/Professional cutting of toenails
	- Knowledge on walking barefooted	
	- Knowledge on preventing foot irritation after washing	Treatment application/Drying
	- Health education knowledge of foot care	Knowledge from attending foot care classes
		Knowledge from health care professionals
		Knowledge from reading handout
	- Foot care knowledge score rate	Good, poor, limited.
Foot care practice/attitude	- The practice of foot inspection	
	- The practice of foot soaking	
	- The practice of washing with soap and warm water	
	- The practice of drying well between toes	
	- The practice of cutting toenails	
	- The practice of wearing socks	
	- Footwear type	Sandal/Leather/Closed toe/Round toe/Sneakers/Slippers, Flipflop/High heel
	- Socks fabrics	Wool/Cotton/Acrylic/Special/Others
	- The practice of walking barefooted	
	- The practice of inspecting footwear	
	- The practice of buying appropriate footwear size	

## Data Availability

Data were obtained from several third party participants and are available upon request from the corresponding authors with the permission of the participants.
